# A Background Search on the Potential Role of *Scutellaria* and Its Essential Oils

**DOI:** 10.1155/2022/7265445

**Published:** 2022-07-27

**Authors:** Mehrukh Zehravi, Chenmala Karthika, Abul Kalam Azad, Zubair Ahmad, Farhat S. Khan, Md. Sohanur Rahman, Rokeya Akter, Md. Habibur Rahman

**Affiliations:** ^1^Department of Clinical Pharmacy Girls Section, Prince Sattam Bin Abdul Aziz University, Alkharj 11942, Saudi Arabia; ^2^Department of Pharmaceutics, JSS College of Pharmacy, JSS Academy of Higher Education and Research, Ooty, Tamil Nadu, India; ^3^Faculty of Pharmacy, MAHSA, Bandar Saujana Putra, 42610, Jenjarom, Selangor, Malaysia; ^4^Unit of Bee Research and Honey Production, Faculty of Science, King Khalid University, P.O. Box 9004, Abha 61413, Saudi Arabia; ^5^Biology Department, Faculty of Sciences and Arts, King Khalid University, Dhahran Al Janoub, Saudi Arabia; ^6^Department of Biochemistry and Molecular Biology, Trust University, Barishal, Ruiya, Nobogram Road, Barishal 8200, Bangladesh; ^7^Department of Pharmacy, Jagannath University, Sadarghat, Dhaka 1100, Bangladesh; ^8^Department of Pharmacy, Southeast University, Banani, 1213 Dhaka, Bangladesh

## Abstract

*Scutellaria* (Lamiaceae), which contains over 350 species, usually known as skullcaps, is found throughout Europe, the United States, and East Asia. In traditional Chinese medicine, several species are used to wipe out heat-evil and remove surface ills (TCM). The current study examines the ethnopharmacology, biological activity, and chemical substances associated with *Scutellaria* species. More than 295 chemicals, including flavonoids and diterpenes, have been identified. *Scutellaria* and its active principles have been shown in studies to have a wide range of pharmacological activities, including antioxidant, antimicrobial, antifeedant, phytotoxic, acaricidal toxicity, antibacterial, anti-inflammatory, and antianalgesic activities. Currently, effective monomeric compounds or active components from *Scutellaria* have been evaluated for pharmacological action *in vivo* and *in vitro*. More data facilitates applications and exploitation of novel medication development.

## 1. Introduction

*Scutellaria* is one of the largest genera within Lamiaceae. *Scutellaria* is a genus that seems to have 360–469 species that could be obtained in Europe, Latin America, Eastern Europe, and Central America. *Scutellaria* species have a strong tradition of usage as herbal remedies for chemotherapies, hepatic and intestinal disorders, asthma, neurological and cardiovascular ailments, and infectious diseases. Furthermore, contemporary pharmacotherapy has substantiated the traditional applications of *Scutellaria* medicinal species, including *Scutellaria lateriflora L*, *Scutellaria barbata D*. Don, and *Scutellaria baicalensis* Georgi [1–3]. This genus has extensive therapeutic potential, encompassing antioxidative, cytotoxic, anti-inflammation, and antivirus qualities, as well as hepatoprotective and neuroprotective abilities. The ongoing evolution of studies on the genus *Scutellaria* revealed parallels and variances in its range, pharmaceutical components, phytochemical constituents, and pharmacological usage. The diversity of *Scutellaria*'s active substance structures and abundant biological properties suggests that illustrative associations between phytochemical analysis, phylogeny, and therapeutic implications (conventional use and advanced pharmacology) are beneficial for investigators to easily recognize the medicinal worth of this genus and investigate and exploit it [4–6].

The Lamiaceae (mint) family comprises 350 species of perennial flowering plants from the genus *Scutellaria*, which is endemic to Asian countries, North America, and Europe [1, 2]. This genus has about 300 species, many of which can be found throughout Asia [3–6]. There are 27 *Scutellaria* species in Iran, 12 of which are endemic [7]. From ancient times, *Scutellaria* genus has been utilized to treat hyperlipidemia, hepatitis, inflammation, allergies, arteriosclerosis, and hypertension [8]. *Scutellaria* has been used for over 2000 years in Asian medicine, notably Chinese medicine, to treat high fever, runny noses, influenza, and high blood pressure [9–12]. *Scutellaria* has anticancer, antibacterial, antioxidant, hepatoprotective, anti-inflammatory, and antiviral properties [8]. *Scutellaria* species are also valuable in the mitigation of nervous system conditions such as phenylethanoid glycosides, insomnia, anxiety, and hysteria [2]. Among the 295 compounds identified in this genus are flavonoids [12] and terpenes (diterpenes, monoterpenes, iridoid glycosides, and triterpenoids) [13].

*Scutellaria* has a high concentration of flavonoids (nearly flavones), which are beneficial chemicals [14]. *S. immaculata* Nevski ex Juz., S. *ramosissima* Popov, and *S. schachristanica* Juz. aerial parts are frequently used to diagnose hypertension, neurodegenerative disorders, and asthma in Uzbekistan. Secondly, they all contain flavonoids and essential oils. Essential oils are secondary plant metabolites that include a wide spectrum of synthetic compounds as well as therapeutic capabilities [15]. They may be harvested from plants in a variety of ways, both classic and cutting-edge [16]. Hydrodistillation, microwave, organic solvent extraction, steam distillation, supercritical CO_2_, ultrasonic, and high-pressure solvent extraction are some of the procedures used to extract essential oils [17, 18]. The components of essential oils are regulated by extraction processes, geographic and climatic conditions, plant storage, physiological age, harvesting time, and drying type. Please keep in mind that the contents of several parts of the plant vary [19–22]. Terpenoids, which are classified as monoterpenes, sesquiterpenes, triterpenes, diterpenes, and tetraterpenes, are the most significant essential oil components [23, 24]. Essential oils protect plants from predators, environmental stress, parasites, and illness, and they also attract insects. They also help in attracting insects to provide an effective reproductive phase [25].

*Scutellaria baicalensis* may be safe to consume by mouth for the vast majority of individuals. It has the ability to induce sleep. Patients who took *Scutellaria baicalensis* had fever and lung irritation. However, there is insufficient information to determine if Scutellaria baicalensis is the cause of these adverse effects. A few also show that some products containing *Scutellaria baicalensis* may cause liver issues in some people. Flavocoxid, a unique combination drug, was determined to be safe in 12-week study studies. However, some people may develop liver issues as a result of this or other combination products. This negative impact does not appear to be prevalent, and it may only be felt by people who have an allergic reaction [11–15].

In this review, we have mentioned the antioxidant, antimicrobial, antifeedant, phytotoxic, acaricidal toxicity, antibacterial, anti-inflammatory, and antianalgesic activities of *Scutellaria* and its essential oils. The current review was compiled using content from the databases Web of Knowledge, Chemical Abstracts, Scopus, PubMed, ScienceDirect, and Google Scholar.

## 2. Traditional Uses

Per this study, around 50 species, 5 subspecies, and 17 variations of *Scutellaria* were utilized using natural remedies (aerial portion, rhizome, or whole plant) in countries like China, Nepal, India, North America, Nepal, Turkey, and Uzbekistan [26, 27]. *Scutellaria* medicinal herbs are being used to treat several diseases, which include peptic diseases (stomach pain, dysentery, bloating), liver function and gall diseases (jaundice, hepatitis), infections (carbuncles, furunculosis), neurological conditions (epilepsy, insomnia, chorea, spasm, hysteria), respiratory problems (respiratory infections, colds, scarlet fever), and traumatic injuries. Also, variability in the pharmacologic portions of *Scutellaria* may be attributed to morphological differences in *Scutellaria* and medication usage choices in various places [27–30]. Conventional herbal remedies in China are created from 32 species and 13 variations, the largest of which are found in the southern region. These medicinal plants' most commonly used sections are the root and, indeed, the entire plant. *Scutellaria* species with fleshy rhizomes are used as medicine in China including *Scutellaria rehderiana*, *Scutellaria baicalensis*, *Scutellaria amoena*, and *Scutellaria viscidula.* Additionally, as per traditional Chinese theory, certain species often have the quality of removing heat, moisture, and poisonous materials, as well as having a significant impact on treating upper respiratory tract infections and acute gastroenteritis [30–34]. *Scutellaria* plants are used whole as remedies; on the other hand, they often have short roots and little plants, such as *Scutellaria barbata*, *Scutellaria galericulata*, *Scutellaria indica*, and *Scutellaria sessilifolia*. These species frequently have the qualities of removing heat, wet, and poisonous elements, boosting blood circulation to alleviate blood stasis, and lowering inflammation to reduce pain, according to traditional Chinese medicine theory [34–36]. Furthermore, certain species, according to traditional Chinese thought, can remove heat, moisture, and harmful elements, as well as have a major influence on curing upper respiratory tract infections and acute gastroenteritis [37–40]. *Scutellaria* plants used as medicines contain short root systems as well as little plants, including *Scutellaria indica*, *Scutellaria barbata*, *Scutellaria galericulata*, and *Scutellaria sessilifolia*. According to the Chinese medicine concept, those species typically get the benefits of eradicating heat, dampness, and poisonous elements, increasing circulation of blood to decrease blood stasis, and decreasing inflammation to ease the pain. They have been shown to treat traumatic injuries, furuncles, and furunculosis and to also reduce inflammation derived from a variety of disorders. *Scutellaria baicalensis* and *Scutellaria barbata* D. are both frequently used natural medicines in China, and they are also used in other Asian countries such as Japan and Korea [40–45].

## 3. Essential Oils

Aromatic plants produce natural volatile oil molecules with a strong odor as bioactive compounds. They are primarily twisted with the use of steam or hydro-distillation, which was initiated in the Middle Ages. Because of their antiseptic, i.e., virucidal, bactericidal, fungicidal, and therapeutic aspects, as well as their scent, they are utilized in embalming, food preparation, and as antibacterial, painkiller, sedative, anti-inflammatory, antispasmodic, and topically anesthetic therapies [45–48]. These characteristics have not altered substantially up to the present day, other than that so much is already understood about all of their modes of action, notably at the antimicrobial level. Essential oils are a crucial component in plant protection throughout nature as antibacterial, antifungal, pesticides, antivirals, and also against herbivores by limiting their hunger for those kinds of plants. They might even invite particular insects to aid in pollen and seed propagation while repelling others. Essential oils are extracted from several fragrant plants that are found in subtropical to warm climates such as those of the Mediterranean and tropical areas when compounds represent a large element of conventional pharmacopeia [48–50]. These were liquid, volatile, clear, but seldom colored, fat-soluble, and common organic solvents, with a density that is generally lower than water [50–55]. There are several approaches to extracting essential oils. The usage of liquid carbon dioxide or microwaves, as a greater or lower pressure distillation using boiling water or heated vapor, is an example of this. Healthcare and culinary usage are now becoming increasingly prevalent as compared to synthetic pharmaceutical goods to safeguard the natural balance due to their bactericidal and fungicidal capabilities. Extraction by steam distillation or expression, for instance, is favored in certain circumstances [55–60]. Extraction with lipid-soluble solvents and, in some cases, supercritical carbon dioxide is preferred for fragrance applications. Thus, the biochemical signature of essential oil products changes not only in the number of molecules extracted but also in the stereochemistry of molecules extracted depending on the mode of extraction, which is decided based on the aim of usage. Climate, age, plant organs, soil composition, and vegetative cycle phase can all improve the level and quantity, as well as proportions of the extracted product. To generate essential oils with a consistent constitution, they must be extracted under such conditions within the same organ of a plant that has grown in the same soil, location, and season. Gas chromatography and mass spectrometry studies are used to chemotype the majority of marketed essential oils. To certify the purity of essential oils, analytical monographs have already been issued (ISO, European pharmacopeia, WHO, Council of Europe) [60–65]. Essential oils have been used for qualities that have already been found in nature, such as antifungal, antibacterial, and insecticidal effects. Approximately 3000 essential oils are currently known, and approximately 300 of them would be economically relevant, particularly in the cosmetic, medicinal, food, agronomic, sanitary, cosmetic, and aroma sectors. Essential oils with their ingredients are applied in aromas and cosmetics, hygiene goods, medicine, horticulture, food custodians and supplements, and organic cures. D-Limonene, d-carvone, and geranyl acetate, for instance, are used in aromas, moisturizers, cosmetics, as fragrance enhancers in food, odors in-home cleaning chemicals, and as lubricating oils [65–68]. Further, essential oils are utilized in massaging as a combination with vegetable oil, in spas, and often in aromatherapy. Essential oils appear to have specific therapeutic characteristics that have been proposed to treat some organ dysfunctions or systemic disorders. Due to the high demand for natural, unadulterated substances in many industries, essential oils are utilized widely throughout the world and are continuing to expand in popularity. As a result, a lot of essential oils are produced around the world to support the cosmetics, aromatherapy, and phytomedicine businesses. Most of them have been efficient substitutes or supplements to added compounds used in the chemical process industries but generally do not get the same side effects [68–73].

## 4. Obtaining Evidence

Far more research reports on the essential oils of several *Scutellaria* species were studied [26–32]. Even though several assessment reviews on the *Scutellaria* genus have been performed [8, 13], knowledge regarding *Scutellaria* essential oils is lacking. As a result, this study focuses on the chemical components and biological activity of essential oils extracted from the *Scutellaria* genus.

## 5. Chemical Constituents

A review of the most current studies on the essential oils of various *Scutellaria* species was conducted [26–32]. While several overview aspects of the *Scutellaria* genus have been undertaken [8, 13], there is little research on *Scutellaria* essential oils. In conclusion, these studies were based on the chemical components and pharmacological effects of essential oils obtained from the genus *Scutellaria*. The concentration of essential oils varies according to the harvesting season, drying conditions, subspecies type, soil pH, geographic location, subspecies type, plant part, and extraction technique [19–22].

**Figure 1:**
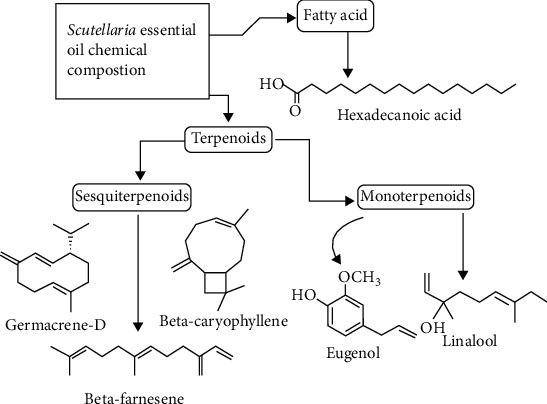
Chemical structures and among the major ingredients found in *Scutellaria* essential oils.

Essential oil compositions are depicted in supplementary file figures a to d; sesquiterpenes are the most abundant component in *Scutellaria* essential oils. The bulk of the essential oils in this species includes *β*-farnesene, hexadecanoic acid, *β*-caryophyllene, germacrene D, linalool, and eugenol.  Figure 1 depicts the structures of these compounds. *Scutellaria* species have been shown to contain a variety of hydrocarbons and oxygenated terpenoid chemicals (Figure 1). Hexadecanoic acid, a saturated fatty acid, is found in plants, animals, and microbes [33]. Germacrene D is a sesquiterpene pioneer of cadences and selinenes [34, 35]. Germacrene D kills mosquitoes, aphids, and ticks [36–38]. *β*-Caryophyllene is a phytocannabinoid-rich sesquiterpene that may help with neuropathic pain, anxiety, endometriosis, ulcerative colitis, and renal safety [39–42]. Linalool is a monoterpene component found in a wide variety of plants that has antinociceptive, antibacterial, and antihyperalgesic properties, but also antibacterial and antifungal action against a variety of pathogens and fungi [43]. Farnesene is a potent pheromone in the vast majority of aphid species [44]. Considering several publications on the essential oils of *Scutellaria* species (over 38), a wide variety of species remain unexplored. Additional investigation into the chemical properties of unreported *Scutellaria* essential oils is thus required [70].

The primary components of *S. diffusa* oil remained revealed to be hexadecanoic acid with 30% and caryophyllene oxide with 9%. Germacrene D with 21%, hexadecanoic acid with 16%, and *β*-caryophyllene with 13% were found identified to be critical elements in S. heterophylla oil. Germacrene D with 40% was perhaps the most important element of *S. salviifolia* oil, preceded by bicyclo germacrene with 14% and *β*-caryophyllene with 4% [70–75].

The essential oils extracted by hydrodistillation from extracts of three *Scutellaria* brevibracteata subspecies (subsp. subverting, subs. brevibracteata, and subsp. Pannosula) from their natural locations in Turkey were all studied simultaneously using gas chromatography (GC) and gas chromatography-mass spectrometry (GC-MS). Depending on the chromatographic study and data analysis, the main determinants in S. brevibracteata subsp. brevibracteata oil were found to be *β*-caryophyllene (22.8%) and caryophyllene oxide (22.8%). The major components of S. brevibracteata subsp. subvelutina oil were 28.3% *β*-caryophyllene, 12.4% linalool, and 10.8% hexadecanoic acid. *S. brevibracteata* subsp. peninsula oil contains high levels of *β*-caryophyllene (36.4%), *β*-cadinol (9.8%), *β*-cadinene (7.0%), and linalool (5.3%). It was, to the best of our knowledge, the first research on the chemical properties of three *Scutellaria brevibracteata* essential oils. TLC, LSC, GLC, and GLC-MS methods were employed to analyze the essential oil *Scutellaria lateriflora* L. (Labiatae) generated by hydrodistillation in northern Iran. In the oil, there were at least 73 different substances. Low quantities of nonterpenoid components were discovered [75–80].

The Lamiaceae family includes *Scutellaria volubilis* and *Lepechinia paniculata*. In Ecuador, they are commonly utilized in the traditional system of medicine. After hydro distillation, the structural activity and chemical characteristics of essential oils derived from aerial parts of *Scutellaria volubilis* in its foliation-blooming period and *Lepechinia paniculata* in its propagation season remained explored. Gas chromatography/mass spectrometry (GC/MS) and gas chromatography/flame ionization detection (GC/FID) approaches were used to examine the composition of these essential oils. The essential oil of *Scutellaria volubilis* was found to have 37 components. Sesquiterpene hydrocarbons are also confirmed to be the primary elements: germacrene D with 20.4%, *β*-caryophyllene with 17.5%, *β*-humulene with 14.7%, and *β*-bisabolene with 5.8%. The essential oil of *L. paniculata* comprised 34 components, the overwhelming of which were sesquiterpene hydrocarbons such as aromadendrene (24.6%), viridiflorene with 12.4%, *β*-selinene with 7.4%, and valencene with 6.7%. Monoterpene hydrocarbons were identified in smaller concentrations, as was *β*-phellandrene with 6.9% and with 7.7%. Including both species, oxygenated monoterpenes and sesquiterpenes constitute less than 5%. This is the first description of this species' chemical characteristics [80–85].

*Scutellaria albida L*. subsp. albida, *Scutellaria albida* L. subsp. Colchica *[Rech.f.]J.R.Edm*., *Scutellaria albida L.* subsp. Condensata [*Rech.f.]J.R.Edm*., and *Scutellaria albida* L. subsp. Velenovskyi [Rech. Linalool with 20% and 29%, respectively, was found recognized as a prominent compound in the oils of S. albida subsp. albida and *S. albida* subsp. condensata. Hexadecanoic acid with 13% was discovered to be a significant constituent in the oil of S. albida subsp. colchica. The oil of *S. albida* subsp. velenovskyi has the highest proportion of -caryophyllene (20%) [85–90].

## 6. Essential Oils and Their Specificities

Essential oils are the main raw material for the aroma and fragrance, food, and pharmaceutical industries. Essential oils are concentrated plant extracts that retain the natural smell and flavor, or “essence,” of their source. Some authors investigated the relationship between lavender essential oil production and gene expression during blooming, aiming to determine the optimum period for essential oil harvest [90]. Other researchers came to the conclusion that changes in chemical variety have a mosaic pattern, which is tied to changes in predator activity and may be influenced by geographic factors or natural enemies. In terms of antigenotoxicity, all of the essential oils assessed showed this preventive effect. Moreover, the manner of defense varied according to the mutagens, i.e., the kinds of defects generated and therefore the kinds of enzymatic identification and activation resulting in translational synthesis or late apoptosis/necrosis [90–95].

## 7. Biological Actions of Scutellaria Essential Oils

### 7.1. Antioxidant Capability

The antibacterial properties of *Scutellaria* genus essential oils are quite low relative to the antioxidant potential of *Scutellaria* extracts [70–72]. Mamadalieva et al. investigated the antioxidant properties of three Uzbek *Scutellaria* species' essential oils (*Scutellaria ramosissima*, *Scutellaria immaculata*, and *Scutellaria schachristanica*). These *Scutellaria* essential oils demonstrated significant antioxidant action due to the incorporation of eugenol, thymol, and carvacrol; however, it was less than ascorbic acid [57]. The antioxidant effect of a Scutellaria baicalensis trihydroxyflavone extract on oxidation generated by UV radiation was investigated using a phosphatidylcholine liposome membrane. The antioxidative activity of baicalin, baicalein, wogonin, and butylated hydroxytoluene (BHT) was also examined as standards [95].

The chemical confirmation of essential oils found from aerial sections of *Scutellaria immaculata Nevski ex Juz*., *Scutellaria ramosissima* M. Pop., and *Scutellaria schachristanica* Juz. (Lamiaceae) rising wild in Uzbekistan was studied using GC and GC–MS. The foremost components of *S. immaculata* essential oils appear to be acetophenone with 30.39%, eugenol with 20.61%, thymol with 10.04%, but also linalool with 6.92%, whereas *S. schachristanica* essential oils appear to be acetophenone with 34.74%, linalool with 26.98%, but also eugenol with 6.92%. Germacrene D with 23.96%, *β*-caryophyllene with 11.09%, linalool with 9.63%, and hexadecanoic acid comprise the oil of *S. ramosissima* with 8.34%. In DPPH, ABTS, and FRAP experiments, the essential oils of *Scutellaria* species displayed lesser antioxidant activity. Especially eugenol showed a significant lowering power in the FRAP experiment (IC_50_ = 2476.9215.8(mM Fe(II)/g)) [95–100].

### 7.2. Antimicrobial Activity

The physiological effects of *Scutellaria* essential oils have been examined, with the majority of the research focusing on antibacterial activity. Eugenol, linalool, and other long-chain alcohols may be responsible for the antibacterial properties of these oils [73]. Antibacterial action may also be aided by other essential oil components such as thymol and alpha-terpineol [74, 75]. Khotimchenko and Yakovleva explored the antibacterial properties of S. barbata essential oils contrary to 17 microorganisms (*Pseudomonas aeruginosa*, *Salmonella paratyphi-A*, *Klebsiella pneumonia*, *Stenotrophomonas maltophilia*, *Serratia marcescens*, *Enterococcus faecalis*, *Staphylococcus aureus*, *Serratia liquefaciens*, *Escherichia coli*, *Staphylococcus haemolyticus*, *Candida tropicalis*, *Staphylococcus simulans*, *Salmonella typhi*, *Staphylococcus epidermidis*, *Citrobacter freundii*, *Shigella flexneri*, and *Candida albicans*) utilizing the disc diffusion and broth microdilution approaches. Because the essential oil had such significant bacteriostatic activity, their data indicated that *S. epidermidis* was perhaps the furthermost sensitive microbe (29 mm inhibition zone and 0.77 mg/mL MBC), whereas *C. albicans* was possibly the most resistant (7-9 mm and 24.50 mg/mL MBC) [69]. The essential oils of *S. strigillosa*, according to Zhu et al., are substantially more efficient against Gram-positive bacteria and fungi than against Gram-negative germs [53]. Thus, according to Pant et al., essential oils of *S. grossa* exhibited antimicrobial properties toward *K. pneumonia*, *E. faecalis*, *B. subtilis*, and *S. enterica* [65]. As per Skaltsa et al., essential oils of *S. rupestris* and *S. sieberi* isolated in Greece had limited potency against S. aureus and B. cereus [28]. Skaltsa et al. discovered that perhaps the essential oil of S. albida subsp albida showed activity against *B. subtilis*, *S. aureus*, *P. aeruginosa*, *E. coli*, and *S. cerevisiae* due to the huge amount of linalool and nerolidol [27]. Dereboylu et al. investigated the antibacterial properties *of S. aureus*, *E. coli*, *S. typhimurium*, *B. subtilis*, *E. faecalis*, and *P. aeruginosa* active ingredients against seven bacteria and one fungus (*S. aureus*, *S. typhimurium*, *E. coli*, *P. aeruginosa*, *E. faecalis*, *B. subtilis*) [58]. The essential oil of *S. repens* being tested evaluated antibacterial activity on *A. tumefaciens*, *E. faecalis*, *K. pneumoniae*, *X. phaseoli*, *S. aureus*, *E. coli*, *S. enterica*, *E. chrysanthemi*, and *P. multocida* [67], where the zone of inhibition for *E. coli* was 23 mm, accompanied by *E. faecalis* at 18 millimeters, *K. pneumonia* at 15 millimeters, and then *B. subtilis* at 12 mm [67].

The plant *Scutellaria barbata* D. Don (Lamiaceae) is endemic to south China. This plant, identified as Ban-Zhi-Lian in Chinese traditional medicine, has been recognized as an antitumor, anti-inflammatory, and diuretic ingredient. *S. barbata* formulations have exhibited leads to growth inhibition in a variety of malignancies. The plant has been applied in the mitigation of digestive system malignancies, lung cancer, breast cancer, hepatoma, and chorioepithelioma in therapy. The plant is expected to possess alkaloids and flavonoids. Polyphenols (apigenin and luteolin) have been identified as bioactive components toward methicillin-resistant Staphylococcus aureus from just a 50% ethanolic leaf extract. E-1-(40-Hydroxyphenyl)-but-1-en-3-one was extracted from a methanolic extraction of the leaves and showed high cytotoxicity in K562 human leukemia cell lines. There is still no published evidence on the chemical components and antibacterial action of *S. barbata* essential oil.

*Scutellaria barbata* essential oil has been produced by hydrodistillation with a 0.3%(*v*/*w*) yield as well as examined by GC and GCMS. Hexahydrofarnesylacetone with 11.0%, 3,7,11,15-tetramethyl-2-hexadecane-1-ol with 7.8%, menthol with 7.7%, and 1-octen-3-ol with 7.7% were the primary components in the oil with 7.1%. The oil's antibacterial efficacy toward 17 microorganisms has been tested through disc diffusion and broth microdilution techniques. Gram-positive bacteria were just more tolerant to the oil than Gram-negative bacteria and yeasts, notably methicillin-resistant *Staphylococcus aureus*.

The aerial portions of three endemic *Scutellaria* species from Lamiaceae are being studied for glandular trichomes shape, volatile content, and antibacterial activity. The examined species have two kinds of glandular trichomes that are morphologically unique. Capitate trichomes were found in all three taxa studied. Peltate glandular trichomes featured a large secretory head with one central and three to eight periphery cells. Even in the petiole of *S. cypria* var. elatior could peltate trichomes found. Steam distillation in a Clevenger type system yielded 0.26-0.47% (*v*/*w*) volatiles and essential oil combinations from three species. The GC-FID and GC-MS methods were used for their studies. By comparing their relative retention indices, mass spectra, and certain literature records, these chemicals were identified. There was a total of 23 identified components. While overall volatile % ages were found to be 99.99%, essential oil yield varies from 34.64 to 92.25% for three species. Trans-caryophyllene with 22.58% and germacrene D with 42.01% were found to be key components of *S. sibthorpii*. Eugenol with 23.05% and palmitic acid with 27.00% were found considered to be significant components of *S. cypria* var. cypria. Furthermore, *S. cypria* var. elatior has a substantial fraction of linalool and palmitic acid, with 10.92% and 46.76%, respectively, where the MIC values of the volatiles towards Gram-negative and Gram-positive bacteria varied from 10 to 20 mg/mL. *C. albicans* was shown to be among the furthermost resistant yeast-like fungus, with MIC values found to be high than 20 mg/mL.

### 7.3. Antifeedant Activity

The essential oils of three *Scutellaria* species (*Scutellaria orientalis* ssp. Alpina, *Scutellaria brevibracteata*, and *Scutellaria hastifolia*) were tested in contradiction to the feeding and egg-laying activities of *Spodoptera littoralis* in research published by Formisano et al. Both *S. brevibracteata* and *S. hastifolia* extraction inhibited female moth egg laying on sheets; however, only *S. hastifolia* essential oil halted *Spodoptera littoralis* larvae from eating on mitigation discs [47]. As per Giuliani et al., the essential oil of *S. rubicunda* subsp. *linnaeana* has antifeedant action in contradiction of *Spodoptera littoralis* [26]. *S. littoralis* is a contraction for the letter S. The essential oil of the plant generated a dose-dependent positive feeding response in littoralis larvae. *Scutalbin* C, *Scutecyprol* B, and *scutecyprol B* remained initiate in aerial sections of *S. rubicunda subsp. linnaeana*. Linnaeana (FI at 100 ppm = 100) was antifeedant to larvae of five Lepidoptera species [1].

### 7.4. Phytotoxic Effect

Phytochemicals from various *Scutellaria* species displayed substantial cytotoxic effects on some human tumor cell lines in vitro. Flavonoids, neoclerodan diterpenoids, iridoids, phenyl alcohol glycosides, and alkaloids have all been identified from *Scutellaria* species. *S. strigillosa* essential oil has been studied for phytotoxicity to amaranth and bluegrass (amaranthus is a worldwide genus of annual or short-lived perennial plants, while bluegrass refers to numerous species of grasses of the genus Poa). Amaranthus seedling progress was completely inhibited by 3 *μ*L/mL essential oil, although bluegrass growth was just slightly affected [53].

### 7.5. Acaricidal Toxicities

The acaricidal toxicities of 1-hydroxynaphthalene from *S. barbata* oil and its derivatives were determined and compared with those of benzyl benzoate. *S. barbata* essential oil has potent acaricidal action than the control sample (benzyl benzoate) [45].

### 7.6. Antibacterial Activity

*S. grossa* essential oil extracts had potent antibacterial effects on *Bacillus subtilis* and *Enterococcus faecalis* (MIC, 31.25–62.5 *μ*L/mL), along with *Klebsiella pneumoniae* as well as *Salmonella enterica* (MIC, 125 *μ*L/mL). *S. lindbergii* Rech.f. ethanolic had to have a considerable antibacterial property, with a MIC value of 6.25 mg/mL^−1^. The steam volatile oil derived from *S. repens aerial* parts displayed identified potential towards *E. faecalis* (the MIC was found to be 125 *μ*L/mL^−1^), *Escherichia coli* (MIC, 31.25 *μ*L/mL^−1^), and *Klebsiella pneumoniae* (MIC, 250 *μ*L/mL^−1^) [56].

The chemical constituents of the steam volatile oil produced by steam distillation of this aerial portion of *Scutellaria grossa* Wall ex Benth. (Lamiaceae) were investigated using capillary GC and GC-MS. Oxygenated monoterpenes were shown to be abundant in the oil (88.6%). There must have been 50 elements discovered, accounting for 94.4% of the overall oil content. Linalool (37.0%) and 1-octen-3-ol (32.0%) were shown to have main components. The oil's antibacterial efficacy towards ten bacterial strains was established by measuring the growth inhibitory zones. The oil had substantial antibacterial action toward Gram-positive *Bacillus subtilis* and *Enterococcus faecalis* bacteria as well as Gram-negative *Klebsiella pneumoniae* but also *Salmonella enterica enterica* bacteria. The minimal inhibitory concentration against *E. faecalis* was reported at 31.25 *μ*L mL^–1^ [56–60].

### 7.7. Anti-inflammatory Action

Many combinations of *Scutellaria baicalensis* hot water extract (SB-HW) and Chrysanthemum morifolium ethanol extract (CM-E) were tested for anti-inflammatory activity. SB-HW (80 g/mL)/CM-E (120 g/mL) or SB-HW (40 g/mL)/CM-E (160 g/mL) substantially decreased LPS-stimulated NO and IL-6 levels in RAW 264.7 cells. SB-HW (80 g/mL)/CM-E (120 g/mL) was shown to be the most effective combination for suppressing MUC5AC secretion in PMA- and LPS-induced NCI-H292 cells. In PMA-induced A549 cells, the active combination also reduced PGE2 and IL-8 production. According to LC-MS/MS tests, the active combination had a high concentration of flavone glycosides such as baicalin and cynaroside. The active combination inhibited phosphorylation of ERK, JNK, and p38 on Western blots, showing that MAPK signaling was inhibited. The active combination, according to our findings, might be employed as a new anti-inflammatory herbal medication [101].

### 7.8. Antianalgesic Activity

*S. edelbergii* crude extract with subfraction analgesic effectiveness was tested in Swiss albino mice at various dosages to treat acetic acid-induced writhes. In this investigation, aspirin was utilized as a control. EtOAc was found to be the most active fraction, with inhibition rates of 37% and 55% at dosages of 50 and 100 mg/kg body weight, respectively, followed by chloroform, with inhibition rates of 29% and 48% at doses of 50 and 100 mg/kg body weight, respectively [102].

## 8. Additional Information

The cytotoxic potential of essential oils found in this prooxidant behavior can make this effective disinfectant and microbial mitigation for personal use, including air purification, personal hygiene, and even internal use via oral ingestion, and also the pesticide approval process for agricultural or supply product preservation [70].

Essential oils have a considerable advantage in that they can be free of long-term genotoxic effects. Furthermore, several of them still have strong antimutagenic potential, which could also be connected to anticarcinogenic action. Current findings have shown that the prooxidant activities of essential oils or certain of their ingredients, as well as those of many polyphenols, are particularly efficient in reducing either tumor volume or tumor growth using apoptotic and/or necrotic activities. Myrica gale essential oil possesses an anticancer effect on lung and colon cancer cell lines, per Carson and Riley [75]. *Nigella sativa* has been established to have an antiproliferative effect and suppressed 1,2-dimethylhydrazine-induced malignancy in rats by Sun et al. Because essential oils have the potential to interact with mitochondrial activity, they would add prooxidant effects and even become true antitumor agents. Several radical-producing drugs are used in anticancer therapies. In the case of essential oils, oxidative generation may be strictly controlled and tailored while representing no harmful or mutagenic risks to healthy cells [44]. Essential oils or related bioactive constituents might be integrated into vectorized liposomes, allowing for more precise quantification. As a result, essential oils may force their way from the classical to the pharmaceutical sphere [76].

*Scutellaria* is a genus of seldom shrubs, herbs, or subshrubs, with a broad range of structures. Many plants in this genus are being used as conventional remedies to cure a variety of ailments all over the world, with their most popular medicinal portions including the root, aerial part, and complete plant. As per the reports, aerial parts of around 12 species, 5 subspecies, and 1 variation have been used in conventional ways. The majority of them will be capable of treating the neurological disorder, trauma, allergies, peptic, cerebrovascular illnesses, hepatic cardiovascular and gallbladder disorders, and malignancy. Likewise, the majority of the therapeutic plants of the genus *Scutellaria* are tiny plants, have short roots, and are present mostly in Europe, Latin America, Asia, and Southwest Asia. The roots of around 16 species and 4 variations are also used to treat a variety of diseases, including respiratory disorders, miscarriage, liver and gall problems, gastric diseases, hypertension, insomnia, and trauma, and are primarily found in China. This species was distinguished by its well-developed rhizome features. Cold symptoms, infectious diseases, snake bites, hepatitis, peptic disorders, gynecologic inflammation, internal injury, renal vacuousness, lumbar discomfort, pyelonephritis, migraine, toothache, major trauma, tinea of the feet and hands, and miscarriage are among the ailments treated with the whole plant. These species, which are mostly perennial plants and subshrubs, are found across East and South Asia. As a result, the identification of therapeutic components may be influenced by vegetation structure and regional medical practices. Moreover, due to its several flavonoids, *Scutellaria* has been deemed a distinct genus of *Scutellarioideae*, and while several chemicals, specifically a number of diterpenes, have already been discovered in *Scutellaria* in recent times. There are also variances in the dispersion of the chemicals based on established chemistry research on these therapeutic plants in the genus *Scutellaria*. Is there a link between many medical components, organic compounds, and applications? As we investigated *S. baicalensis* and *S. barbata*, the two largest studied species in terms of nutrients, we determined the difference in chemical structure, pharmacological components, and therapeutic properties [66]. *Scutellaria baicalensis* is an herbaceous plant with a dense and meaty rhizome, and the dried root of *Scutellaria baicalensis* has an abundance of 4′‑deoxyflavones, which lead to *Radix Scutellariae*'s clarifying warmth and detoxing properties. *S. barbata*, on hand, is a perennial herb with a small and thick rhizome that comprises flavonoids and is high in neo-clerodane diterpenoids, and the entire plant has been used to treat cancer, major injuries, and carbuncles [55–60]. Neo-clerodane diterpenoids are recognized to be reflective components of *S. barbata*. Moreover, they are the existing research area of *S. barbata* with varied geometries and strong carcinogenic and antifeedant actions. Hence, it is suggested that every species' different molecular components coincide with unique medical value and that their pharmacological purposes correlate with their morphological features. Ignoring the fact that so much research on their chemical characteristics is concentrated on the pharmacological parts, and various bioactive components have been separated from *Scutellaria* medicinal plants, their impact on the plants' claimed medical benefit or demonstrable pharmacological properties has not yet been satisfactorily studied. Furthermore, phytochemistry research on nearly 50% of those phytochemical constituents has not yet been completed. As a result of insufficiently defined studies on these herbs and shrubs, the links between the therapeutic component chemical characteristics and traditional usage in other medicinal belonging to the genus *Scutellaria* require future investigation. More studies need to be done to look for particular bioactive components in *Scutellaria* plants [44]. *Scutellaria* is a Lamiaceae genus that has been used as medicine for countless generations [76, 77]. Multiple investigations into the essential oils of several *Scutellaria* species have now been conducted [8, 13, 26–32]. Nonetheless, because several species of the *Scutellaria* genus will not be researched, many studies on the composition and biologically active compounds of underresearched *Scutellaria* essential oils may be completed. The present investigation highlights the chemical characteristics and bioactivities of the *Scutellaria* genus (antioxidant, antifeedant, antibacterial, phytotoxic, and acaricidal activities). The essential characteristics comprised *β*-farnesene, hexadecanoic acid, *β*-caryophyllene, germacrene D, linalool, and eugenol (any of these oil constituents contain therapeutic capabilities). This article can be used as a guide in the disciplines of essential oils and ethnopharmacology.

## 9. Conclusion

Essential oils are secondary metabolites with a variety of organic structures that have therapeutic activity depending on their content. With about 250 species, *Scutellaria* is a Lamiaceae genus of perennial plant species. For a long time, it has been used to treat hypertension, arteriosclerosis, allergies, hyperlipidemia, inflammatory illnesses, hypertension, and hepatitis. Several substances, particularly essential oils, have been discovered in numerous studies on the chemical constituents of the *Scutellaria* genus. Various compounds have been discovered in studies on the chemical compositions of essential oils from the *Scutellaria* genus. Chemical constituents and biological activities of *Scutellaria* essential oils were identified. The main components of this genus' essential oils are hexadecenoic acid, farnesene, caryophyllene, germacrene D, linalool, and eugenol. Although 38 studies on the essential oils of *Scutellaria* species are still available, there appears to be a large number of species that need to be investigated. As a result, more research is needed into the various elements and pharmacological actions of unstudied *Scutellaria* essential oils. Based on available data, this study examined studies on the chemistry and biochemical processes of *Scutellaria* essential oils, including phytotoxic, antioxidant, antimicrobial, antifeedant, and antiparasitic toxic effects.

## Data Availability

No associate data is present in this review. Although if necessary, corresponding author will update.

## Supplementary Materials

Supplementary Materials Figure 1: the relative abundance of sesquiterpene hydrocarbons in *Scutellaria* species essential oils. Figure 2: the relative abundance of monoterpene hydrocarbons in *Scutellaria* species essential oils. Figure 3: the relative abundance of oxygenated sesquiterpenes in *Scutellaria* species essential oils. Figure 4: the relative abundance of oxygenated monoterpenes in *Scutellaria* species essential oils.
