# Supplementary material for: A Background Search on the Potential Role of Scutellaria and Its Essential Oils
Source: Biomed Res Int. 2022 Jul 27;2022:7265445. doi: 10.1155/2022/7265445 (PMC9365597; doi:10.1155/2022/7265445)
Supplement: Supplementary Materials — Figure 1: the relative abundance of sesquiterpene hydrocarbons in Scutellaria species essential oils. Figure 2: the relative abundance of monoterpene hydrocarbons in Scutellaria species essential oils. Figure 3: the relative abundance of oxygenated sesquiterpenes in Scutellaria species essential oils. Figure 4: the relative abundance of oxygenated monoterpenes in Scutellaria species essential oils. [file 7265445.f1.docx]

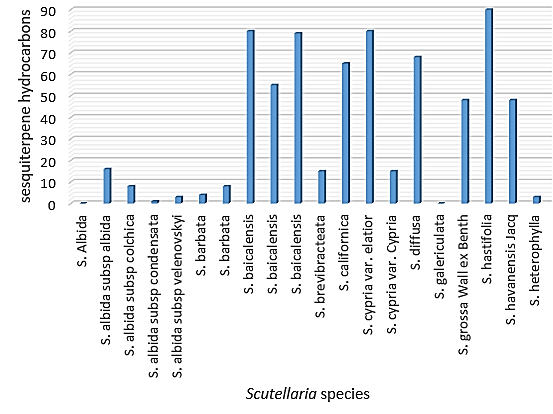

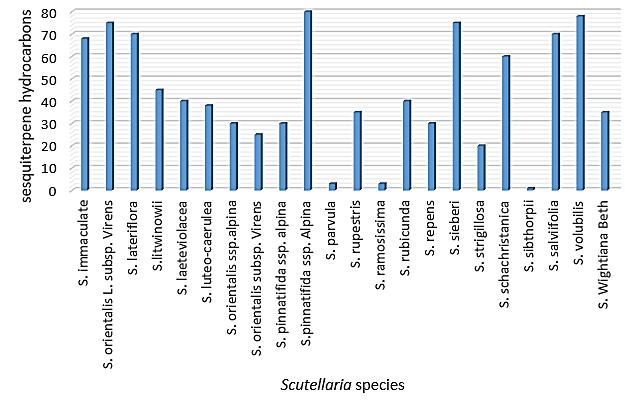


Figure 1: The relative abundance of sesquiterpene hydrocarbons in *Scutellaria* species essential oils.


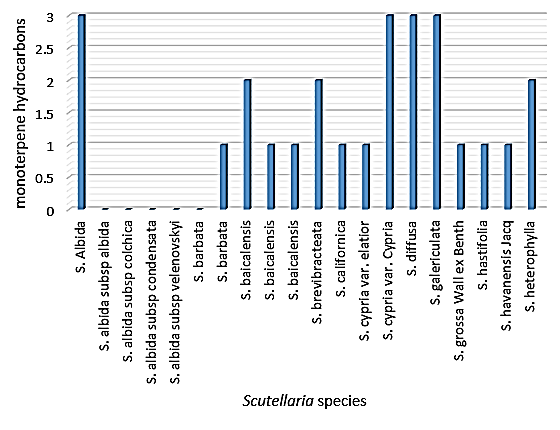

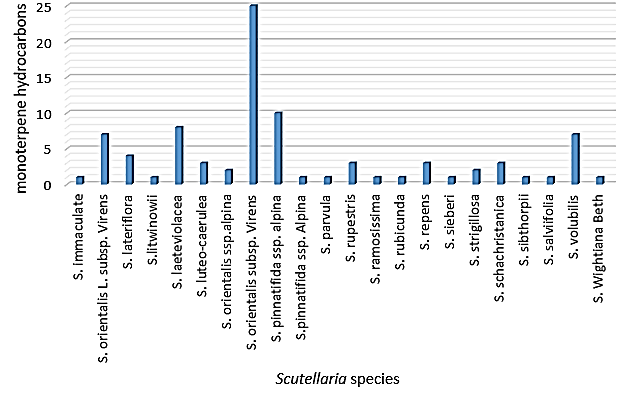


Figure 2: The relative abundance of monoterpene hydrocarbons in *Scutellaria* species essential oils.


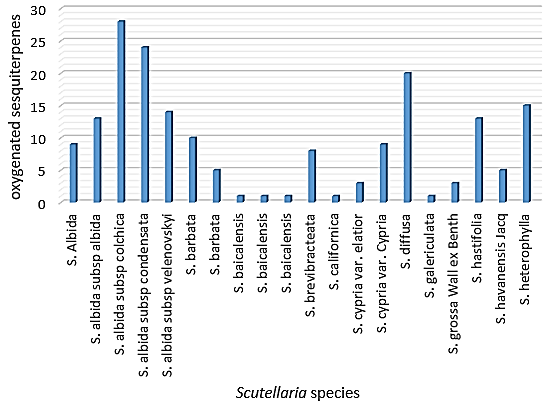


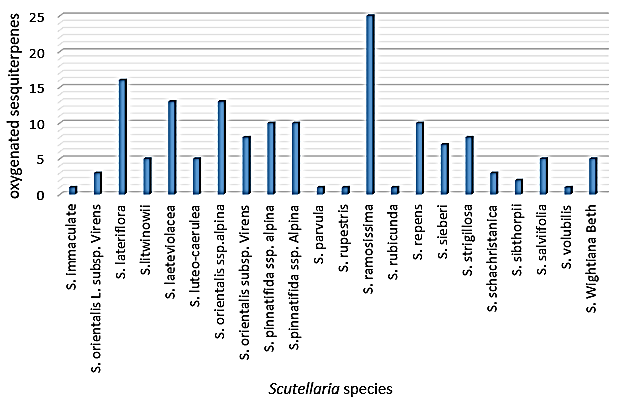


Figure 3: The relative abundance of oxygenated sesquiterpenes in *Scutellaria* species essential oils.


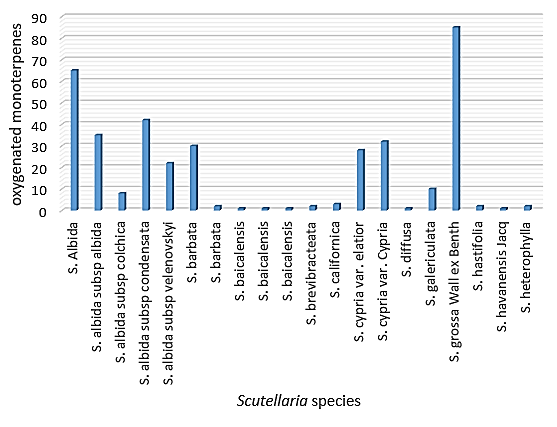


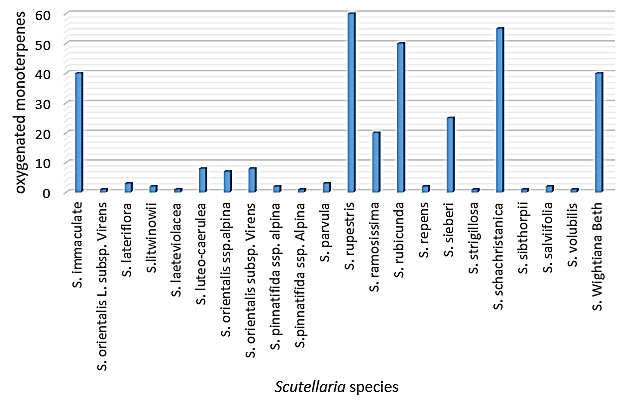


Figure 4: The relative abundance of oxygenated monoterpenes in *Scutellaria* species essential oils.
